# Derivation of normal macrophages from human embryonic stem (hES) cells for applications in HIV gene therapy

**DOI:** 10.1186/1742-4690-3-24

**Published:** 2006-04-19

**Authors:** Joseph S Anderson, Sriram Bandi, Dan S Kaufman, Ramesh Akkina

**Affiliations:** 1 Department of Microbiology, Immunology and Pathology, Colorado State University, Fort Collins, Colorado 80523, USA; 2 Department of Medicine, University of Minnesota Medical School, Minneapolis, MN 55455, USA

## Abstract

**Background:**

Many novel studies and therapies are possible with the use of human embryonic stem cells (hES cells) and their differentiated cell progeny. The hES cell derived CD34 hematopoietic stem cells can be potentially used for many gene therapy applications. Here we evaluated the capacity of hES cell derived CD34 cells to give rise to normal macrophages as a first step towards using these cells in viral infection studies and in developing novel stem cell based gene therapy strategies for AIDS.

**Results:**

Undifferentiated normal and lentiviral vector transduced hES cells were cultured on S17 mouse bone marrow stromal cell layers to derive CD34 hematopoietic progenitor cells. The differentiated CD34 cells isolated from cystic bodies were further cultured in cytokine media to derive macrophages. Phenotypic and functional analyses were carried out to compare these with that of fetal liver CD34 cell derived macrophages. As assessed by FACS analysis, the hES-CD34 cell derived macrophages displayed characteristic cell surface markers CD14, CD4, CCR5, CXCR4, and HLA-DR suggesting a normal phenotype. Tests evaluating phagocytosis, upregulation of the costimulatory molecule B7.1, and cytokine secretion in response to LPS stimulation showed that these macrophages are also functionally normal. When infected with HIV-1, the differentiated macrophages supported productive viral infection. Lentiviral vector transduced hES cells expressing the transgene GFP were evaluated similarly like above. The transgenic hES cells also gave rise to macrophages with normal phenotypic and functional characteristics indicating no vector mediated adverse effects during differentiation.

**Conclusion:**

Phenotypically normal and functionally competent macrophages could be derived from hES-CD34 cells. Since these cells are susceptible to HIV-1 infection, they provide a uniform source of macrophages for viral infection studies. Based on these results, it is also now feasible to transduce hES-CD34 cells with anti-HIV genes such as inhibitory siRNAs and test their antiviral efficacy in down stream differentiated cells such as macrophages which are among the primary cells that need to be protected against HIV-1 infection. Thus, the potential utility of hES derived CD34 hematopoietic cells for HIV-1 gene therapy can be evaluated.

## Background

Human embryonic stem cells (hES cells) show great promise for many novel cellular therapies due to their pluripotent nature [1]. These cells have the capacity to give rise to mature cells and tissues that arise from all three germ layers during embryonic development [2-4]. Several pluripotent hES cell lines have so far been derived from the inner cell mass of human blastocysts and can be cultured indefinitely in an undifferentiated state [5-7]. Thus, these cells provide a renewable source of pluripotent stem cells from which many types of differentiated cells could be produced for experimental and therapeutic purposes. Cell differentiation protocols currently exist for the derivation of neurons, cardiomyocytes, endothelial cells, hematopoietic progenitor cells, keratinocytes, osteoblasts, and hepatocytes to name a few [2,3,8,9]. In addition to providing for potential cellular replacement therapies, opportunities exist in programming hES cells to correct a genetic defect and/or to express a therapeutic transgene of interest. Using such approaches, many possibilities exist for treating a number of genetic and immune system disorders [1].

Many novel applications can be foreseen for hES cells in infectious disease research. AIDS is a potential disease that can benefit from exploiting hES cells for cell replacement therapy as they have the capacity to differentiate into various hematopoietic cells. HIV continues to be a major global public health problem with infections increasing at an alarming rate [10,11]. Given the present lack of effective vaccines and the ineffectiveness of drug based therapies for a complete cure, new and innovative approaches are essential. Gene therapy through intracellular immunization offers a promising alternative approach and possible supplement to current HAART therapy [12-14]. HIV mainly targets cells of the hematopoietic system, namely, T cells, macrophages, and dendritic cells [15]. As infection progresses, the immune system is rendered defenseless against other invading pathogens and succumbs to opportunistic infections. There is a great deal of progress in the area of stem cell gene therapy for AIDS [12]. A primary goal of many ongoing studies is to introduce an effective anti-HIV gene into hematopoietic stem cells [16-18]. As these cells possess the ability to self renew, they have the potential to continually produce HIV resistant T cells and macrophages in the body thus providing long term immune reconstitution. These approaches use CD34 hematopoietic stem cells for anti-HIV gene transduction via integrating viral vectors such as lentiviral vectors [16-18]. Lentiviral vectors have several advantages over conventional retroviral vectors since higher transduction efficiencies can be obtained and there is less gene silencing. The CD34 cells currently used for many therapies are primarily obtained from bone marrow or mobilized peripheral blood [1,19]. Thus, CD34 progenitor cells are an essential ingredient for HIV gene therapy.

In view of the need for CD34 cells for HIV gene therapy as well as for other hematopoietic disorders, if one can produce these cells in unlimited quantities from a renewable source, it will overcome the limitations of securing large numbers of CD34 cells for therapeutic purposes. In this regard, progress has been made in deriving CD34 cells from hES cells (hES-CD34). Different methods currently exist to derive CD34 cells from hES cells with varying efficiencies [20-27]. Recent reports have indicated the capacity of hES cell derived CD34 cells to give rise to lymphoid and myeloid lineages thus paving the way for utilization of these cells for hematopoietic cell therapy [20,27-29].

For the effective utilization of hES-CD34 cells for HIV gene therapy, a number of parameters need to be examined. First, one has to demonstrate that hES-CD34 cells can give rise to macrophages and helper T cells which are the main cells that need to be protected against HIV infection. Recent evidence has shown that hES-CD34 cells can give rise to myelomonocytic cells [21]. However, thorough phenotypic or functional characterization of these cells is lacking. It is also not clear if these cells are susceptible to HIV infection. Similarly, although the hES-CD34 cells were shown to have lymphoid progenitor capacity, only B cell and natural killer (NK) cell differentiation has been examined so far [21,28]. The capacity to generate T cells remains to be evaluated. With this background, as a first step, our primary goal in these studies is to examine the capacity of hES-CD34 cells to give rise to phenotypically and functionally normal macrophages and whether such cells are susceptible to productive HIV infection. Since lentiviral vectors have been shown to successfully transduce hES cells [30-33], we further investigated the ability of transduced hES cells to differentiate into transgenic macrophages that can support HIV-1 infection. Demonstration of HIV-1 productive infection in these cells will permit future efficacy evaluations of anti-HIV genes in this system. Here we show that normal and lentiviral vector transduced hES-CD34 cells can give rise to phenotypically and functionally normal macrophages that support HIV infection thus paving the way for many novel approaches to evaluate their potential for HIV gene therapy.

## Results

### Derivation of macrophages from hES cells

**Figure 1:**
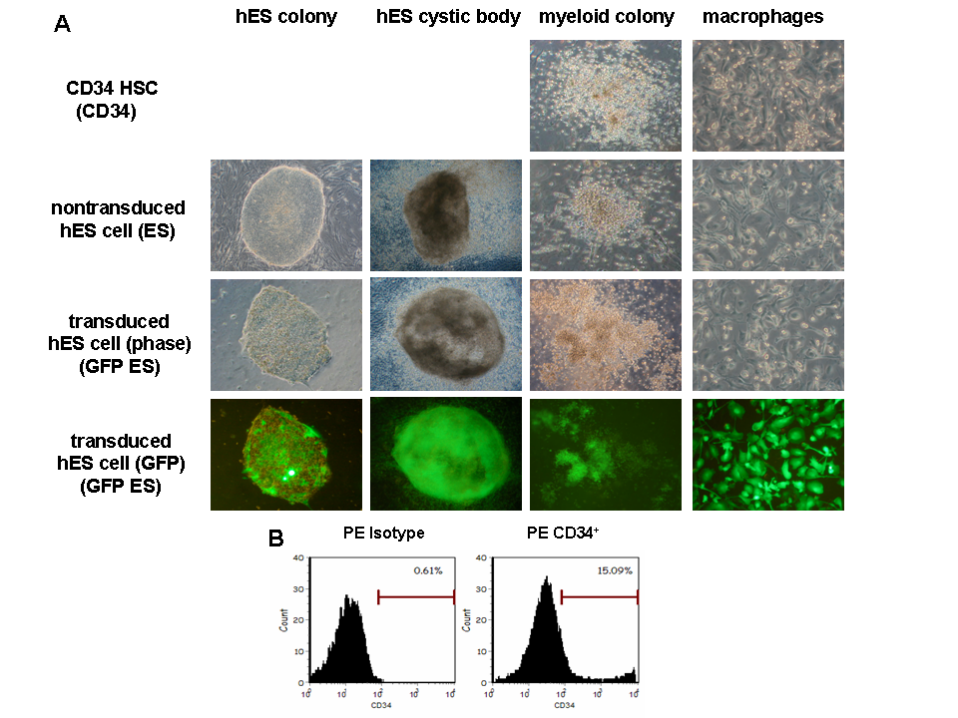
**Derivation of macrophages from lentiviral vector transduced and normal hES cells**. A) Transduced and non-transduced H1 hES cells were cultured on mouse S17 bone marrow stromal cell layers to derive cystic bodies. Cystic body derived CD34 cells were purified by positive selection with antibody conjugated magnetic beads and placed in methocult media to obtain myelomonocytic colonies. Pooled colonies were cultured in liquid cytokine media supplemented with GM-CSF and M-CSF to promote macrophage growth. For comparison, fetal liver derived CD34 cells were cultured similarly to derive macrophages. Representative ES cell colonies, cystic bodies, methocult colonies, and derivative macrophages are shown with GFP expressing cells fluorescing green under UV illumination. B) Representative FACS profile of hES cell derived CD34 cells stained with PE conjugated antibodies. Percent positive CD34 cells are shown with isotype control shown in the left panel.

**Figure 2:**
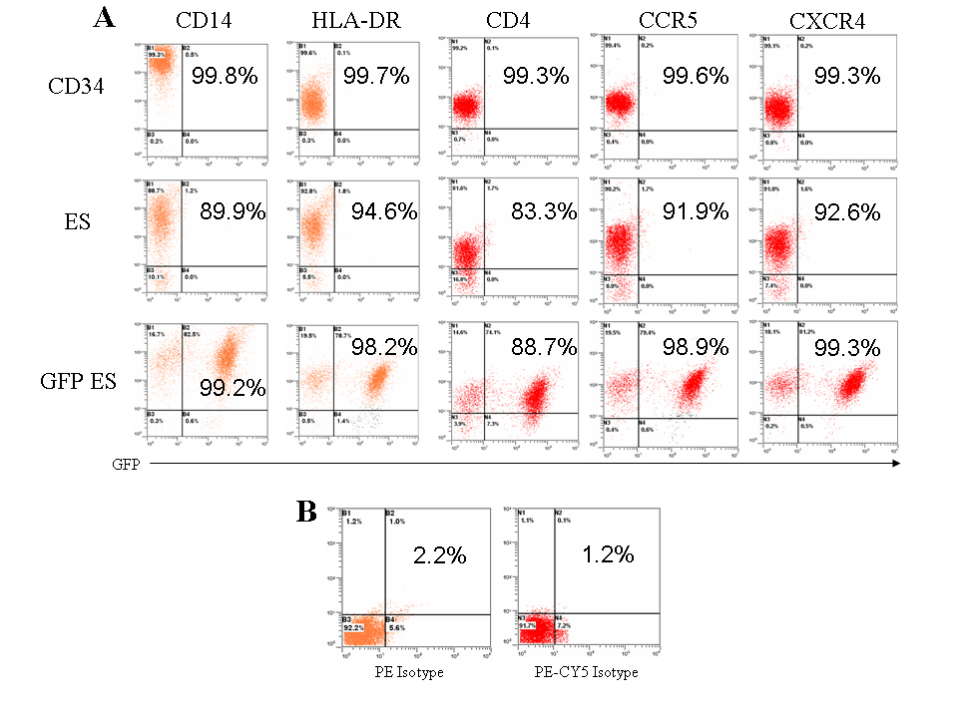
**Phenotypic FACS analysis of hES cell derived macrophages**. A) Macrophages derived from transduced and nontransduced hES CD34 and fetal liver CD34 cells were stained with antibodies to CD14, HLA-DR, CD4, CCR5, and CXCR4 and the expression of these surface markers was analyzed by FACS. B) Isotype controls for PE and PE-CY5 antibodies. Percent positive cells are displayed in the plots for each respective cell surface marker staining. Dot plots are representative of triplicate experiments.

Undifferentiated hES cell colonies grown in media supplemented with 4 ng/ml bFGF displayed normal morphology of pluripotent human embryonic stem cells with tight and discreet borders on the MEF feeder layers (Fig 1A). Similarly, lentiviral vector transduced hES cell colonies, also displayed normal morphology and growth characteristics (Fig 1A). As expected, the vector transduced colonies displayed green fluorescence due to the presence of the GFP reporter gene. When cultured on irradiated S17 mouse bone marrow stromal cells, both nontransduced and transduced hES cells developed into embryonic cystic bodies (Fig 1A). FACS analysis of single cell suspensions of the cystic bodies showed levels of CD34 cells which ranged from 7–15%. Figure 1B displays a representative FACS profile of hES-CD34 cells. Purified CD34 cells were later cultured in semi-solid methylcellulose medium to derive myeloid colonies. Both nontransduced (denoted as ES in figures) and vector transduced (denoted as GFP ES in figures) hES cell derived CD34 cells gave rise to normal myelomonocytic colonies similar to human fetal liver derived CD34 cells (denoted as CD34 in figures) (Fig 1A). When pooled colonies were cultured further in liquid cytokine media for 12–15 days for differentiation, the cells developed into morphologically distinct macrophages (Fig 1A). When compared, the morphology of macrophages derived from all stem cell progenitor populations appeared similar. These results were found to be consistent in replicative experiments. The transgene GFP expression was also maintained during the differentiation of hES cells into mature macrophages. GFP expression in cystic body derived CD34 cells was around 80% (data not shown) with similar levels seen in differentiated macrophages (Fig 2).

### hES cell derived macrophages display a normal phenotypic profile

Macrophages play a critical role in immune system function and are also major target cells for many viral infections including HIV-1. Distinct surface phenotypic markers exist on these cells and, thus far, there has been no thorough evaluation of hES cell derived macrophages. Therefore we analyzed hES cell derived macrophages for the presence of characteristic cell surface markers and compared these to the phenotypic profile displayed on fetal CD34 cell derived macrophages. The surface markers analyzed were CD14, a monocyte/macrophage specific marker, HLA-DR (a class II MHC molecule found on antigen presenting cells), CD4, the major receptor for HIV-1 infection, and CCR5 and CXCR4, chemokine receptors which are critical coreceptors essential for HIV-1 entry. EGFP expression was also analyzed to determine the levels of transduction and any transgene silencing that may occur during differentiation. Fetal liver (CD34), nontransduced (ES), and vector transduced (GFP ES) hES cell derived macrophages were all positive for the monocyte/macrophage marker CD14 (99.3%, 88.7%, and 99.2%, respectively) (Fig 2A). However, the mean fluorescent intensity (MFI) was found to be lower on hES cell derived macrophages. Surface expression of HLA-DR was observed at similar levels between macrophages derived from fetal liver CD34 cells (99.6%), nontransduced hES cells (92.8%), and transduced hES cells (98.2%) (Fig 2A). CD4 levels were comparable for all stem cell derived macrophages (99.2%, 83.3%, and 88.7%, respectively) (Fig 2A). CCR5 and CXCR4 cell surface expression was also observed for fetal liver CD34 cell (99.6% and 99.3%), nontransduced hES cell (91.9% and 92.6%), and transduced hES cell (98.9% and 99.3%) derived macrophages (Fig 2A). As compared to fetal liver CD34 cell derived macrophages, hES cell derived macrophages displayed a higher level of expression of CXCR4. Isotype controls for both PE and PECY5 stains are shown in Fig 2B. The above phenotypic data are representative of triplicate experiments.

### Transgenic hES cell derived macrophages are functionally normal

**Figure 3:**
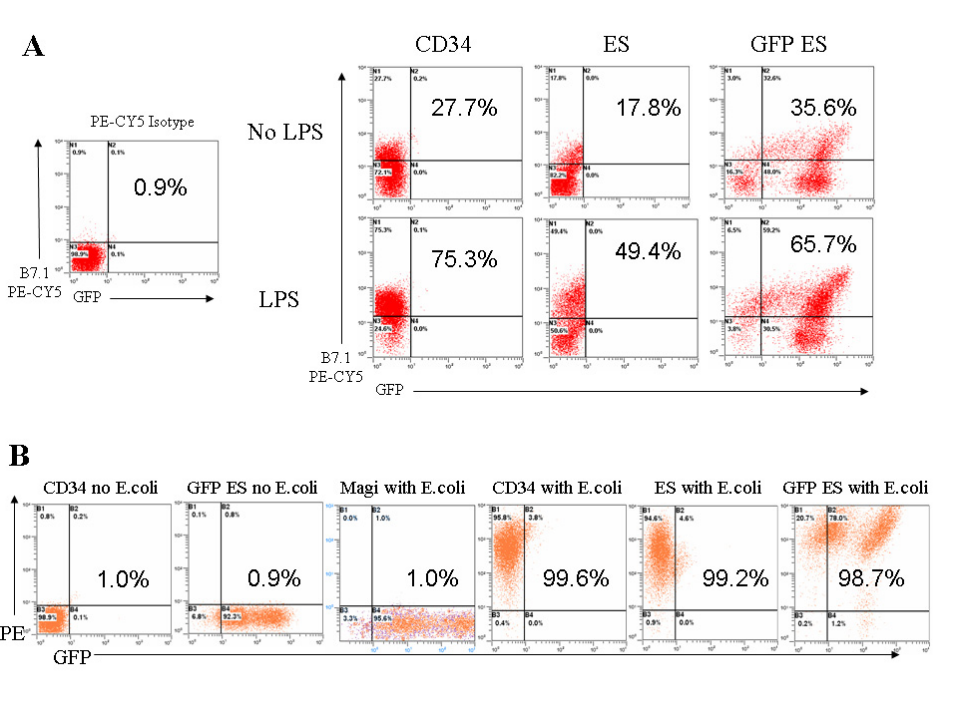
**Functional analysis of hES cell derived macrophages for B7.1 costimulatory molecule upregulation and phagocytosis of E. coli particles**: A) Mature macrophages were stimulated with LPS to determine B7.1 upregulation. Twenty-four hours post-stimulation, macrophages were labeled with a PE-CY5 conjugated anti-B7.1 antibody and analyzed by FACS. B7.1 upregulation data are representative of triplicate experiments. Isotype control is shown in the left panel. B) To assess phagocytic function, *E. coli *Bioparticles^® ^were added directly to the cultured macrophages. Twenty four hours post-addition, cells were analyzed by FACS. Percent positive cells are displayed in the plots for each experiment. These data are representative of triplicate experiments.

The antigen presenting cell surface specific marker HLA-DR (MHC II) on normal macrophages is critical for presenting antigen to CD4 T cells. A second co-stimulatory molecule, B7.1 is present at low basal levels on resting macrophages and is necessary to activate T cells. Its expression is elevated upon activation with certain stimuli such as LPS. Our results of LPS stimulation of respective macrophages have shown upregulation of B7.1 with values for fetal liver CD34 cell (CD34) (27.9% to 75.4%) nontransduced (ES) (17.8% to 49.4%) and transduced (GFP ES) (35.6% to 65.7%) hES cell derived macrophages (Fig 3A). These values represent a significant upregulation of B7.1 for all three macrophage populations.

Another important function of macrophages is their ability to phagocytose foreign material and present antigenic peptides on their cell surface. To evaluate phagocytic function, fluorescently labeled *E. coli *Bioparticles^® ^were added to macrophage cultures followed by FACS analysis. Nontransduced (94.6%) as well as lentiviral vector transduced (98.7%) hES cell derived macrophages were found to be capable of phagocytosing the Bioparticles^® ^in comparison to fetal liver CD34 cell derived macrophages (95.8%) (Fig 3B). These values are representative of triplicate experiments. Magi-CXCR4 cells with no phagocytic capacity were used as non-phagocytic cell controls and similarly exposed to *E. coli *Bioparticles^® ^(Fig 3B). No uptake of the bacteria could be seen. Thus, uptake of *E. coli *Bioparticles^® ^by macrophages is indicative of active ingestion.

**Figure 4:**
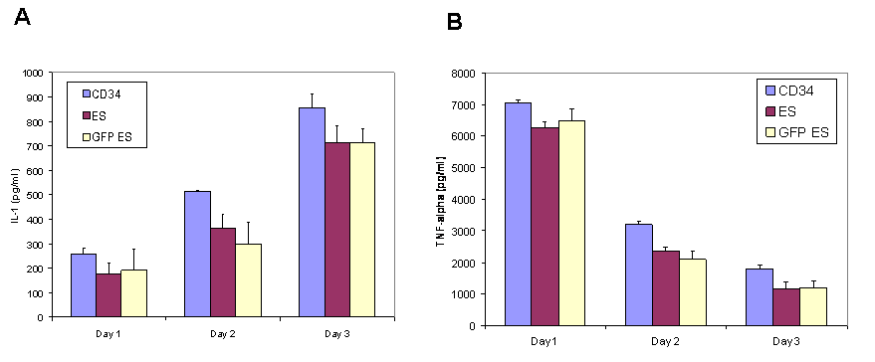
**Cytokine IL-1 and TNFα secretion by stimulated hES cell derived macrophages**: Macrophages derived from transduced and nontransduced hES and fetal liver CD34 cells were stimulated with 5 μg/ml LPS. On days 1, 2, and 3 post-stimulation, supernatants were collected and assayed by ELISA for (A) IL-1 and (B) TNFα. Experiments were done in triplicate.

Macrophages, as effector cells, play a key role in the inflammatory response. Activated macrophages secrete various cytokines, two of the major ones being IL-1 and TNF-α. To determine if hES cell derived macrophages have such a capacity, cells were stimulated with LPS. On days 1, 2, and 3 post-stimulation, culture supernatants were analyzed by ELISA to detect IL-1 and TNF-α. As seen in figure 4A, there were no significant differences in IL-1 secretion between the three sets of macrophages. Similarly, nontransduced and transduced hES cell derived macrophages were also capable of TNF-α secretion upon LPS stimulation. However, levels of the respective cytokines detected were slightly lower than those from fetal liver CD34 cell derived macrophages (Fig 4B). The values of cytokine secretion levels represent triplicate experiments.

### hES cell derived macrophages support productive HIV-1 infection

The above data have shown that hES cell derived macrophages are very similar to normal human macrophages based on phenotypic and functional analysis. In addition to being important cells of the immune system, macrophages are among the major target cells for certain viral infections, particularly for HIV-1. We wanted to determine if hES cell derived macrophages were susceptible to HIV-1 infection compared to standard macrophages. In these studies, we only used an R5-tropic strain of HIV-1 since macrophages are natural targets for this virus. Our results from challenge studies of these cells clearly indicated the capacity of hES cell derived macrophages in supporting a productive infection. Levels of virus increased up to 15 days similar to non-hES derived macrophages showing that the initial viral input was amplified in productive viral infection. However, the levels of viral yield were found to be slightly lower for the ES cell derived macrophages. In the case of GFP-ES macrophages, there was a decline in viral titer. This could be due to possible lower numbers of cells present in the initial cultures.

## Discussion

As a first step towards the use of hES cells for hematopoietic stem cell and HIV gene therapies, we have shown here that phenotypically and functionally normal macrophages could be derived from hES-CD34 cells. Both non transduced and lentiviral vector transduced hES cells were found to be capable of generating CD34 cells that give rise to macrophages which could support productive HIV-1 infection. Current sources of CD34 cells consist of human bone marrow, cytokine mobilized peripheral blood, fetal liver, and cord blood [34]. However, the number of cells that can be obtained for manipulations is not unlimited. Therefore, deriving CD34 cells for therapeutic and investigative purposes from hES cells with unlimited growth potential has the advantage of a consistent and uniform source.

The ability to obtain phenotypically normal and functionally competent macrophages from hES cells is important to evaluate their potential therapeutic utilities in the future. Additionally, testing of transgenic hES cells derived via lentiviral vector gene transduction is also helpful to determine the stability of the transgene expression and their capacity for differentiation into end stage mature cells such as macrophages. Based on these considerations, both non- transduced and lentiviral vector transduced hES cells were evaluated for their capacity to give rise to CD34 progenitor cells. In colony forming assays using semisolid methylcellulose medium, the morphology of myelomonocytic colonies derived from hES CD34 cells appeared similar to that of fetal liver CD34 cells. When subsequently cultured in cytokine media that promotes macrophage differentiation, morphologically normal macrophages were obtained with hES-CD34 cells similar to that of fetal liver CD34 cells. At higher magnification, the macrophages displayed flat projecting cellular borders with fried egg appearance with distinct refractory lysosomal granules in the cytoplasm (data not shown). Lentiviral vector transduced hES cells also did not display any abnormal growth or differentiation characteristics as compared to nontransduced hES-CD34 cells indicating no adverse effects due to vector integration and expression. Transduced cells gave rise to cystic bodies with similar CD34 cell content and profiles upon development. The transduced hES-CD34 cells also gave rise to apparently normal macrophages that expressed the transgene as shown by GFP expression. These results are consistent with those of others that showed normal differentiation of hES cells to other cell types following lentiviral transduction [32].

A requirement for successful cellular and HIV-1 gene therapy is that mature end stage cells derived from CD34 progenitor cells be phenotypically and functionally normal to maintain and restore the body's immunological function. Accordingly, hES cell derived macrophages were evaluated to determine if they met these criteria. Macrophages display distinct cell surface markers upon end stage differentiation. To determine whether hES cell derived macrophages display these surface markers, FACS analysis was performed to detect the presence of CD14, HLA-DR (MHCII), CD4, CCR5, and CXCR4. As observed in Fig 2A, both nontransduced and transduced hES cell derived macrophages expressed all of these markers with some differences in their levels of expression. HLA-DR, CD4, and CCR5 expression profiles were comparable between all cell types analyzed. Even though all cell types analyzed stained positive for CD14, relative expression of CD14 was slightly lower on hES cell derived macrophages compared to fetal liver CD34 cell derived macrophages. On the contrary, the levels of CXCR4, a chemokine receptor involved in cellular homing, were found to be higher on hES-CD34 cell derived macrophages. This may be due to inherent differences in the cell types and/or due to their physiological state at the time of harvest [35]. Additional hES cell lines need to be evaluated in the future to establish if these differences are consistent. A major functional role of macrophages *in vivo *is their ability to serve as professional antigen presenting cells. During this process macrophages present antigen peptide fragments complexed with both classes of MHC molecules and deliver a costimulatory signal through the expression of B7 molecules. Upon stimulation with LPS, hES-CD34 cell derived macrophages had shown upregulation of the costimulatory molecule B7.1 similar to cells derived from fetal liver. Furthermore, the hES-CD34 cell derived macrophages also showed a normal capacity to ingest foreign particles in phagocytosis assays using *E.coli *Bioparticles^®^. In addition to antigen presentation and phagocytosis, macrophages also play a critical role in inflammation and secrete cytokines in response to external stimuli. When exposed to LPS, the hES-CD34 cell derived macrophages secreted two important cytokines IL-1 and TNF-α similar to that of fetal liver derived cells.

The above data has established that phenotypically and functionally normal macrophages could be derived from hES-CD34 cells. Macrophages in addition to playing important physiological roles are also major cell targets for certain viral infections, particularly HIV-1. Here we evaluated the susceptibility of hES-CD34 cell derived macrophages to be productively infected with HIV-1. Similar to that of fetal liver CD34 cell derived cells, the hES-CD34 macrophages also supported HIV-1 infection although the levels of viral yield differed somewhat. However this should not be a major concern for testing anti-HIV genes in these cells. In all the above experiments, the vector transduced transgenic macrophages also behaved similarly to that of nontransduced cells showing that they were also physiologically normal. The lack of vector toxicity on cellular maturation is encouraging for future work with transduced hES-CD34 cells to derive other important differentiated cells like T cells and dendritic cells relevant for HIV studies.

Although there are numerous studies on hES cell differentiation into many important end stage mature cells, systematic work on hES cell hematopoietic differentiation and thorough characterization of end stage mature cells that participate in critical immune responses has just begun [21,27-29]. Our current results established that physiologically normal macrophages could be derived from hES cells and that these cells have the potential for use in cellular and gene therapies. To our knowledge this is the first demonstration that hES cell derivatives can be used for infectious disease research. Due to the extensive ability for hES cells to self-renew, large numbers of differentiated cells can be derived so that infection studies and evaluation tests can be carried out in a more standardized way.

Our results showing that both normal and transgenic derivative macrophages support HIV-1 infection points out to their utility for testing anti-HIV constructs transduced into hES-CD34 cells and pave the way for their application in stem cell based HIV gene therapy. So far a number of studies including our own have tested many gene therapeutic constructs in CD34 cells from conventional sources. These constructs include anti-HIV ribozymes, RNA decoys, transdominant proteins, bacterial toxins, anti-sense nucleic acids, and most recently siRNAs [36-50]. In addition, a number of cellular molecules that aid in HIV-1 infection such as cellular receptors and coreceptors CD4, CCR5 and CXCR4 have also been successfully tested in CD34 cell derived macrophages and T cells [16,18,38]. Some of these approaches have progressed into clinical evaluations as well [14,51,52]. Based on our current results, many of these novel anti-HIV constructs can also be tested in hES-CD34 cells for their potential application.

Although there are advantages of using hES cell derived CD34 cells for potential cellular therapies, transplantation of these cells constitutes an allogenic source with immune rejection as a major issue. However, a recent study using human leukocyte reconstituted mice suggested that hESCs and their derivative cell types were less prone to invoking an allogeneic response [53]. Other recent studies demonstrated successful engraftment of primary and secondary recipients with hES cell derived hematopoietic cells in both immunodeficient mice and in vivo fetal sheep models adding further support that any obstacles could be overcome [23,54,55]. Moreover, multiple novel strategies to avoid immune-mediated rejection of hES cell-derived cells have been proposed [56,57]. It is not too far in the future that even autologous hES cells may be derived from specific individuals for deriving CD34 cells which can be used for cell replacement therapy.

## Conclusion

Phenotypically normal and functionally competent macrophages could be derived from hES-CD34 cells. Since these cells are susceptible to HIV-1 infection, they provide a uniform source of macrophages for viral infection studies. Based on these results, it is also now feasible to transduce hES-CD34 cells with anti-HIV genes such as inhibitory siRNAs and test their antiviral efficacy in down stream differentiated cells such as macrophages which are among the primary cells that need to be protected against HIV-1 infection. Thus, the potential utility of hES derived CD34 hematopoietic cells for HIV-1 gene therapy can be evaluated.

## Materials and methods

### Growth, propagation and lentiviral transduction of hES cells

The NIH approved human ES H1 cell line was obtained from WiCell (Madison, Wisconsin). hES cell colonies were cultured on mouse embryonic fibroblasts (MEF) (Chemicon, Temecula, CA) in the presence of DMEM-F12 (Invitrogen, Carlsbad, CA) supplemented with 20% KNOCKOUT serum replacement with 1 mM L-glutamine, 1% Nonessential Amino Acids, 0.1 mM β-mercaptoethanol, 0.5% penicillin/streptomycin, and 4 ng/ml human basic fibroblast growth factor. Culture medium was replaced daily with fresh complete DMEM-F12. Mature colonies were subcultured weekly by digesting with collagenase IV as previously described [5]. A VSV-G pseudotyped lentiviral vector (SINF-EF1a-GFP) containing a GFP reporter gene (kindly supplied by R. Hawley, George Washington University) was used for hES cell transductions as previously described (30, 58). Generation of the pseudotyped vector in 293T cells and its concentration by ultracentrifugation were described previously [30,48]. For vector transduction, the undifferentiated hES cells were prepared into small clumps of 50–100 cells with enzyme digestion as done for routine passaging of cells. The cell clumps were incubated with the vector for 2 hrs in the presence of polybrene 6 ug/ml. A secondary cycle of transduction was done by adding fresh vector and incubating for another 2 hrs. The general vector titers were 1 × 10^7 ^and the multiplicity of infection was 10. The transduction efficiency was about 50%. The transduced colonies were cultured on MEF like above.

### Derivation and purification of CD34 cells from hES cells

Undifferentiated hES cells were cultured on S17 mouse bone marrow stromal cell monolayers to derive cystic bodies containing CD34+ hematopoietic progenitor stem cells. hES cell cultures were treated with collagenase IV(1 mg/ml) for 10 minutes at 37°C and subsequently detached from the plate by gentle scraping of the colonies. The hES cell clusters were then transferred to irradiated (35 Gy) S17 cell layers and cultured with RPMI differentiation medium containing 15% FBS (HyClone, Logan, UT), 2 mM L-glutamine, 0.1 mM β-mercaptoethanol, 1% MEM-nonessential amino acids, and 1% penicillin/streptomycin. Media was changed every 2 to 3 days during 14–17 days of culture on S17 cells [20].

After allowing adequate time for differentiation, hES cystic bodies were harvested and processed into a single cell suspension by collagenase IV treatment followed by digestion with trypsin/EDTA supplemented with 2% chick serum (Invitrogen, Carlsbad, CA) for 20 minutes at 37°C. Cells were washed twice with PBS and filtered through a 70 uM cell strainer to obtain a single cell suspension. To assess the levels of CD34 cells in the bulk cell suspension, cells were labeled with PE conjugated anti-CD34 antibody (BD Biosciences, San Jose, CA) and analyzed by FACS. To purify the CD34 cells, Direct CD34 Progenitor Cell Isolation Kit (Miltenyi Biotech, Auburn, CA) was used following the manufacturer's protocol. Isolated CD34 hematopoietic progenitor stem cells were then analyzed by FACS as mentioned above to determine cell purity. For comparative experiments, human CD34 hematopoietic progenitor cells were also purified from fetal liver tissue as described above.

### Derivation of macrophages from hES cell derived and human fetal CD34 cells

CD34 cells were cultured initially in semisolid media to derive myelomonocytic colonies followed by liquid culture in cytokine supplemented media as described below. Purified CD34+ progenitor cells (~2.5 × 10^5 ^to 4.0 × 10^5^) were placed directly into Methocult semisolid medium (Stem Cell Technologies, Vancouver, BC), mixed, and cultured in 35 mm plates. Myeloid colonies were allowed to develop for 12–15 days. Upon differentiation and proliferation, myelomonocytic colonies were harvested by the addition of 5 ml DMEM containing 10% FBS, 10 ng/ml each GM-CSF and M-CSF. Cells (~10^6^) were placed in a 35 mm well and allowed to adhere for 48 hours. At two and four days post-harvest, medium was replaced with fresh complete DMEM supplemented with 10 ng/ml GM-CSF and M-CSF. By 4–5 days, cells developed into mature macrophages which were used for subsequent phenotypic and functional characterization.

### Phenotypic analysis of hES cell derived macrophages

To determine if nontransduced and lentiviral vector transduced hES cell derived macrophages display normal macrophage surface markers, FACS analysis was performed using respective fluorochrome conjugated antibodies. Fetal liver derived CD34+ cells as well as nontransduced and transduced hES cell derived macrophages were evaluated in parallel. Cells were scraped from their wells, washed two times with PBS, and stained with the following antibodies: PE-CD14, PE-HLA-DR, PECY5-CD4, PECY5-CCR5, PECY5-CXCR4 (BD Biosciences, San Jose, CA). A blocking step was first performed by incubating the cells with the respective isotype control for 30 minutes at 4C before staining with the respective cell surface marker antibodies. Isotype control staining was used to determine background levels. FACS analysis was performed on a Beckman-Coulter EPICS ^® ^XL-MCL flow cytometer with data analysis using EXPO32 ADC software (Coulter Corporation, Miami, FL). A minimum of 8,000 cells were analyzed in each FACS evaluation.

### Functional analysis of hES cell derived macrophages

Physiological roles of macrophages include phagocytic and immune related functions. To determine if hES cell derived macrophages were functionally normal, a stimulation assay to determine upregulation of the costimulatory molecule B7.1 was performed. Activated macrophages upregulate the expression of B7.1 upon activation with various stimuli. Accordingly, fetal liver CD34, nontransduced hES, and GFP-alone transduced hES cell derived macrophages were stimulated by the addition of LPS (5 ug/ml) to the cell culture medium. Twenty-four hours post-stimulation, cells were stained with an anti-B7.1 antibody labeled with PE-Cy5 (BD Biosciences, San Jose, CA) and analyzed by FACS. To assess the hES cell derived macrophages' phagocytic function, 5 ug/ml of fluorescently labeled *E. coli *Bioparticles^® ^(Invitrogen, Carlsbad, CA) were added directly to the cell culture medium. Four hours later, macrophages were washed six times with PBS and fresh medium with 10 ng/ml GM-CSF and M-CSF was added. Twenty-four hours later, cells were analyzed by FACS for the presence of ingested Bioparticles^® ^which can be detected in the PE (FL2) channel. Lentiviral vector transduced Magi-CXCR4 cells, a HeLa cell derivative with no phagocytic capacity, were used as non-phagocytic cell controls and similarly exposed to *E. coli *Bioparticles^®^

Human ES cell derived macrophages were also analyzed for their ability to secrete two major cytokines, IL-1 and TNF-α, upon external stimulation. Accordingly, macrophages were stimulated with 5 ug/ml of LPS during culture. On days 1, 2, and 3 post-stimulation, cell culture supernatant samples were collected and analyzed by a Quantikine^® ^ELISA kit (R&D Systems, Minneapolis, MN). Non-stimulated supernatants were also analyzed for basal levels of cytokine secretion.

### HIV-1 infection of hES cell derived macrophages

To determine if hES cell derived macrophages can be infected with HIV-1 and support viral replication, cells were challenged with a macrophage R5-tropic BaL-1 strain of HIV-1. An m.o.i. of 0.01 in the presence of 4 ug/ml polybrene was used. At different days post-infection, culture supernatants were collected and assayed for p24 antigen by ELISA. To quantify viral p24 levels, a Coulter-p24 kit (Beckman Coulter, Fullerton, CA) was used.

## Competing interests

The author(s) declare that they have no competing interests.

## Authors' contributions

JA and SB contributed equally to this work. SB was responsible for deriving CD34 cells from the hESC and culturing macrophages. JA performed the phenotypic, functional and infection assays on the differentiated macrophages. DSK provided hES cell protocols and supplied lentiviral vector transduced cells. RA was responsible for the overall experimental design and implementation of the project.

**Figure 5:**
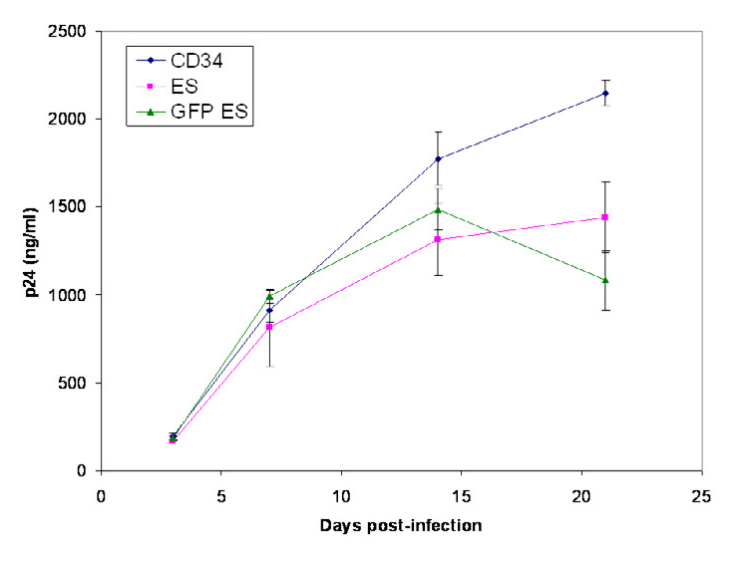
**hES cell derived macrophages support productive HIV-1 infection**: Macrophages derived from transduced and nontransduced hES CD34 and fetal liver CD34 cells were infected with macrophage R5-tropic HIV-1 BaL-1 strain at an m.o.i. of 0.01. Culture supernatants were collected on different days post infection and assayed for viral p24 antigen by ELISA. Data is representative of triplicate experiments.
